# Antimicrobial efficiency and cytocompatibility of resveratrol and naringin as chemical decontaminants on SLA surface

**DOI:** 10.1128/spectrum.03679-23

**Published:** 2024-09-06

**Authors:** You Zhou, Zhe Shen, Yan Xu, Xin-na Qian, Wei Chen, Jing Qiu

**Affiliations:** 1 Department of Oral Implantology, Affiliated Stomatological Hospital of Nanjing Medical University, Nanjing, China; 2 State Key Laboratory Cultivation Base of Research, Prevention and Treatment for Oral Diseases, Nanjing, China; 3 Jiangsu Province Engineering Research Center of Stomatological Translational Medicine, Nanjing, China; Center de Biologie Integrative, Toulouse, France

**Keywords:** antimicrobial efficiency, cytocompatibility, resveratrol, naringin, chemical decontaminant

## Abstract

Bacterial biofilms are the major etiology agent of peri-implant disease. Chemical decontamination is a promising treatment strategy against bacterial biofilms; however, its applications are limited by its low efficiency and poor biocompatibility. In contrast to three conventional cleaners (sterile saline, hydrogen peroxide, and chlorhexidine), this study used resveratrol and naringin solutions to remove mature *Staphylococcus aureus* and *Porphyromonas gingivalis* biofilm on sandblasted (with large grit and acid-etched (SLA) titanium surface. To determine changes in surface characteristics, the surface wettability and roughness were measured, and micromorphology was observed by scanning electron microscopy. With crystal violet (CV) and live/dead bacterial staining, residual plaque quantity and composition were measured. The biocompatibility was tested using pH and cytotoxicity, as well as by osteoblasts (MC3T3-E1) adhesion, proliferation, and differentiation, and fibroblasts (L-929) proliferation were also analyzed. It was found that resveratrol and naringin solutions were more effective in restoring surface characteristics and also showed that less plaque and viable bacteria were left. Naringin removed *S. aureus* biofilms better than chlorhexidine. Alkaline resveratrol and naringin solutions increased cell adhesion, proliferation, and osteogenic differentiation without any cytotoxicity. Resveratrol increased the expression of mRNA and protein associated with osteogenesis. In conclusion, resveratrol and naringin effectively restored SLA titanium surface characteristics and decontaminated the biofilm with good biocompatibility, suggesting their therapeutic potential as chemical decontaminants.

**IMPORTANCE:**

Bacterial biofilms are considered the primary etiology of peri-implant disease. Physical cleaning is the most common way to remove bacterial biofilm, but it can cause grooving, melting, and deposition of chemicals that alter the surface of implants, which may hamper biocompatibility and re-osseointegration. Chemical decontamination is one of the most promising treatments but is limited by low efficiency and poor biocompatibility. Our study aims to develop safer, more effective chemical decontaminants for peri-implant disease prevention and treatment. We focus on resveratrol and naringin, two natural compounds, which have shown to be more effective in decontaminating biofilms on dental implant surfaces and exerting better biocompatibility. This research is groundbreaking as it is the first exploration of natural plant extracts’ impact on mature bacterial biofilms on rough titanium surfaces. By advancing this knowledge, we seek to contribute to more effective and biocompatible strategies for combating peri-implant diseases, enhancing oral health, and prolonging implant lifespan.

## INTRODUCTION

In recent years, peri-implant disease has become more prevalent, which is mainly manifested by mucositis of the surrounding tissues and progressive bone loss around the implant, ultimately resulting in implant failure (1). Bacterial biofilms are considered the primary etiology of the peri-implant disease (2). Through a comparison of the bacterial biofilms found in both healthy and diseased conditions, it was shown that *Staphylococcus aureus* and *Porphyromonas gingivalis* were present in higher abundances on the surface of the implant in cases of peri-implantitis (3, 4). Hence, the primary objective of peri-implant disease therapy is to eliminate biofilms from the titanium surface (5).

The predominant method for eliminating bacterial biofilm from titanium implant surfaces is by physically cleaning the surface, such as the use of curettes, air powder polishers, ultrasonic scalers, titanium brushes, and other similar tools (2). Despite undergoing physical cleaning, biofilms on the titanium surface remained partially intact, particularly in hard-to-reach deep and narrow areas that are inaccessible to equipment tips (6, 7). Moreover, the rough surface formed after physical cleaning continues to retain bacteria in the grooves and pits and facilitates further bacterial adhesion (7). Physical cleaning can also cause grooving, melting, and deposition of chemicals that alter the surface of implants, which may hamper biocompatibility and re-osteointegration (2).

The prevention and treatment of peri-implant diseases can be achieved through chemical decontamination (8). Chemical debridement can penetrate and clean hard-to-reach areas, effectively breaking down the existing plaque structure. A retrospective clinical investigation demonstrated that the combination of mechanical treatment with chemotherapeutic agents was much more effective than mechanical treatment alone in managing peri-implantitis (9). Topically applied antimicrobial agents like sterile saline (SS), hydrogen peroxide (HP), chlorhexidine (CHX), or citric acid can facilitate biofilm removal on titanium surfaces (8), and it was concluded that both citric acid and chlorhexidine had a better cleaning effect (10). However, citric acid tends to erode and disintegrate the protective layer of oxide on the metal surface. This process not only impairs its biocompatibility but also leads to cytotoxic effects in the nearby healthy tissue (11–13). Currently, used chemical agents are not very effective in eliminating microbes and can cause bio-safety related damage. Therefore, it is imperative to carry out more research studies to explore novel chemotherapy agents.

Recent studies confirm the antibacterial properties of natural plant extracts. Resveratrol (RES), a natural polyphenolic antioxidant, is widely found in plants such as grapes, blueberries, and cranberries, as well as in red wine. It has shown that moderate resveratrol can inhibit the growth of *S. aureus*, *Enterococcus faecalis*, and *Pseudomonas aeruginosa* and also inhibit biofilm formation of *Aeromonas hydrophila*. (14–16). In addition, naringin (NAR), a flavonoid glycoside extracted from citrus fruits (17), has been shown to resist bacterial and fungal growth, such as *Listeria monocytogenes*, *Escherichia coli*, and *S. aureus*, and also showed potential for inhibiting biofilm (18–20). Even though natural extracts from plants have significant antibacterial activity against planktonic bacteria, no studies have been conducted on their ability as chemical decontaminants to remove mature plaque biofilms from rough titanium surfaces.

The objective of this study was to identify novel chemical decontaminants that were highly effective, safe, and non-resistant to prevent and treat peri-implant diseases. The efficiency of resveratrol and naringin solution was examined on the morphology of the surface, removal of bacterial biofilms, and cytocompatibility of sandblasted with large grit and acid-etched titanium (SLA-Ti) disks compared with three commonly used chemotherapeutic agents, including 0.9% sterile saline, 3% hydrogen peroxide, and 0.12% chlorhexidine.

## MATERIALS AND METHODS

### Solution and specimen preparation

Preparation of chemotherapeutic agents: resveratrol (100 mg; 99% purity; Sigma, Shanghai, China) and naringin (100 mg; 98% purity; Sigma, Shanghai, China) crystal powder were dissolved separately in 5 mL of anhydrous ethanol with sufficient stirring. The volume was then adjusted to 100 mL by adding deionized water (ddH_2_O) to obtain a 0.1% solution of RES (wt/vol) and a 0.1% solution of NAR (wt/vol). To prepare a 0.12% CHX (wt/vol), 0.12 mL of 20% CHX (Macklin, Shanghai, China) was diluted in 20 mL ddH_2_O. A commercially available 0.9% SS (Leagene, Shanghai, China) and 3% HP (Nanguo, Guangdong, China) were used in the study. To ensure sterility, all solutions were filtered in the biosafety cabinet with 0.22 µm bacterial filters (KURABO, Japan) and preserved in the sealed container at 4°C before use.

Preparation of SLA-Ti surfaces: three different sizes (Φ5 mm × 1 mm, Φ10 mm × 1 mm, and Φ30 mm × 1 mm) of commercially pure titanium (Grade 4; Purity 99.5 wt%; Baoji Titanium, China) were grounded and polished sequentially with silicon carbide (Sic) sandpaper (400, 600, 800, 1,200, and 1,500 grit). After sandblasting with Al_2_O_3_ particles at 0.4 MPa, the titanium disks were etched in HF/HNO_3_ without heating for 10 min, then immersed in H_2_SO_4_/HCl at 80°C for 45 min. The titanium disks underwent a sequential cleaning process using ultrasonic treatment in ddH_2_O, 75% ethanol, and ddH_2_O, with each cleaning phase lasting 15 min. The disks were then disinfected in an autoclave at 121°C for 15 min and dried in an oven at 65°C. A total of 498 SLA-Ti disks were prepared in this study, including 192 pieces of 5 mm diameter, 294 pieces of 10 mm diameter, and 12 pieces of 30 mm diameter.

### Bacterial incubation and biofilm growth

*S. aureus* (ATCC 25923) was grown in Luria-Bertani (LB; Kinghunt, Jiangsu, China) broth in an aerobic chamber (Shel Lab G16-Z, Shanghai, China) at 37°C for 24 h. Hemin at 0.001% (Dulai Biological, China) and vitamin K1 at 0.0001% (Aladdin, China) were added to the brain heart infusion (BHI, OXIOD, United Kingdom). *P. gingivalis* (ATCC 33277) was dispersed in it and placed in the anaerobic chamber (RUskinn Bugbox, England) which was set at 10% H_2_, 5% CO_2_, and 85% N_2_ at 37°C for 48 h.

In the logarithmic growth phase, *S. aureus* was diluted to 1 × 10^6^ CFU/mL with fresh LB, while *P. gingivalis* was diluted to 1 × 10^8^ CFU/mL with fresh BHI by measuring the bacterial density using a turbidity meter (WGZ-2XJ, Xinrui, Shanghai, China). For biofilm formation, 288 pieces of 10 mm diameter SLA-Ti disks were separated equally into two categories, and each disk was placed in a single well of sterile 48-well plates, then 500 µL of bacterial suspension (LB + *S. aureus* or BHI + *P. gingivalis*) was added to each well. Afterward, the disks in the *S. aureus* suspension were incubated for 24 h in the aerobic chamber, while the disks in the *P. gingivalis* suspension were incubated for 48 h in the anaerobic chamber. Following incubation, the culture medium was aspirated with a pipette, keeping the mature biofilm on the surface of the specimen. The SLA-Ti disks with *S. aureus* biofilm (SLA-Sau) and SLA-Ti disks with *P. gingivalis* biofilm (SLA-Pg) were obtained.

### Decontamination procedures

One hundred twenty pieces of SLA-Sau and 120 pieces of SLA-Pg were randomly classified into five groups, respectively, and placed in new 48-well plates. Five different chemical debridement agents were used to treat contaminated samples including 0.9% SS, 3% HP, 0.12% CHX, 0.1% RES, and 0.1% NAR. The chemotherapeutic agents, 500 µL each, were carefully introduced into the 48-well plates by pouring them down the sides of the wells. The sample was submerged for 1 min to replicate the clinical procedure of treating dental implants and was rinsed once with phosphate-buffered saline (PBS; Gibco, United States). The positive controls for the experiment were composed of SLA-Sau and SLA-Pg without decontamination, whereas the negative controls consisted of uncontaminated SLA-Ti disks.

### Surface characterization

The contact angle, defined as the angle at the interface where water, air, and solid meet, measures the surface’s wettability by water. Low contact angles indicate a tendency of water to spread and adhere to the surface, while high contact angles suggest the surface’s propensity to repel water (21). After drying the disks in an oven at 37°C for 30 min, the wettability of titanium disks with different chemical debridement was evaluated by the contact angle meter (Kino, United States), with SLA-Sau, SLA-Pg, and SLA-Ti disks as controls. ddH_2_O (2 µL) was dropped onto each specimen’s surface at five random points, then the contact angles were recorded. In addition, the roughness of each sample was measured at five random points using the optical profilometer (MicroXamTM, PhaseShift, USA). All tests were performed in triplicate.

For performing the microscopic analysis, different titanium disks were fixed with 2.5% glutaraldehyde in a new 48-well plate at 4°C for 2 h, then rinsed with PBS. All titanium disks were subjected to a series of dehydration steps for 15 min with ethanol at various concentrations, including 30%, 50%, 70%, 80%, 90%, and 100%. After sputter-coated with gold, the residual bacterial biofilm and surface morphology were observed by scanning electron microscope (SEM; Ultra 55, Zeiss, Germany) under two magnifications, 5,000× and 20,000×, at 10.0 kV and a working distance of 5 mm. In each group, three different samples were observed.

### Biomass quantification analysis

The biofilm present on the sample after decontamination was examined via CV staining. Specimens were carefully transferred to another 48-well plate, fixed by adding 500 µL of 4% paraformaldehyde (biosharp, Beijing, China) for 15 min, and then dried at a temperature of 37°C. Each well was supplemented with 500 µL of 0.01% CV [(wt/vol), Biosharp, Beijing, China) and kept at room temperature for 5 min. The wells were then washed with PBS and decolorized using 95% ethanol. The well plates were placed in a shaker (SK-O330-Pro, DLAB Scientific, CA, United States) for 30 min at 100 rpm. A new 48-well plate was then filled with 500 µL of the mixture, which was pipetted from each well. A microplate spectrophotometer (Spectramax190, MD, United States) was used to determine the biofilm biomass at an optical density (OD) of 595 nm. This experiment consisted of three samples per group and was repeated thrice.

A bacterial quantification assay was performed after decontamination procedures using the LIVE/DEAD BacLight Bacterial Viability Kit (L7012, Invitrogen, United States) with a laser scanning confocal fluorescence microscope (LSCM; LSM710, Zeiss, Germany). To prepare the live/dead stain, 30 µL of SYTO 9 (excitation = 480 nm, emission = 500 nm) and 30 µL of propidium iodide (PI; excitation = 490 nm, emission = 635 nm) were diluted in 20 mL of ddH_2_O. For each sample, 500 µL of the dye mixture was added at room temperature for 20 min in the dark. Next, the excess dye was removed by rinsing with PBS. The LSCM was then used to evaluate each disc positioned on a glass slide with magnifications of 200×. For this assay, three duplicates were performed. The mean fluorescence intensity ratio and fluorescence area of live and dead bacteria in each LSCM image were analyzed using ImageJ (NIH, United States).

### Cell culture

The MC3T3-E1 osteoblast cells and L-929 fibroblast cells were obtained from the Cell Bank of the Chinese Academy of Sciences (Shanghai, China). The α-Minimum Essential Medium (Gibco, United States) and Dulbecco’s Modified Eagle’s Medium (Gibco, United States), which contained 10% fetal bovine serum (Gibco, United States) and 1% penicillin/streptomycin (Gibco, United States), were used for the culturing of MC3T3-E1 and L-929 cells. The cells were incubated in a humidified atmosphere composed of 5% CO_2_ and 95% air at 37°C. A two-day cycle of medium renewal was followed. Cell passages were performed at the 80% cell confluence, and the fourth generation was used in the series.

### Acidity determination

The acidity of different chemical rinses was measured by an electronic pH detector (S975, Mettler Toledo, Shanghai, China). SS, HP, CHX, RES, and NAR were dispensed into the glass test tubes, the dry clean electrode of the pre-calibrated pH meter was immersed in each solution, and the values were recorded. Measurements were repeated three times for each solution.

### Cytotoxicity assay

MC3T3-E1 and L-929 cells were both seeded at the density of 3 × 10^3^ cells/well of 96-well plates, respectively, and placed in an incubator for 24 h. A mixed solution was prepared by adding 10 µL of each of the five different chemical agents to 100 µL of the medium. This solution was then applied separately to the plates for 1 min, followed by rinsing twice with PBS. Subsequently, using the CCK-8 kit (Beyotime, China), 100 µL of fresh medium and 10 µL of reagent were added and incubated in the dark for 2 h at 37°C. Next, 100 µL of the solution was aspirated from each well and transferred to a new 96-well plate, and the absorbance OD value (λ = 450 nm) of each well was measured by the microplate spectrophotometer. A culture medium containing CCK-8 reagent was used as blank control, and the following formula was utilized for the calculation of cell viability:


$$Cell\;Viability\;\left(\%\right)=\frac{\left(A_{Sample}-A_{Blank}\right)}{\left(A_{Control}-A_{Blank}\right)}\times 100\%.$$

The experiment was performed in triplicate, with each group consisting of five wells.

### Cell adhesion assay

SLA-Sau was rinsed using different chemical decontaminations, followed by washing with PBS. The osteoblasts were inoculated at 5 × 10^3^ cells/well on the surface of specimens in the 48-well plates for 4 h. After washing thoroughly with PBS, each sample was fixed via 4% paraformaldehyde for 10 min at room temperature. Rhodamine phalloidin (Cytoskeleton, United States) was then applied to stain cells for 30 min, keeping the plate in the dark. After incubation, 4′,6-diamidino-2-phenylindole (Beyotime, Shanghai, China) was added for 60 s. Fields on each sample were selected randomly using the LSCM at 200× and 400× magnifications. MC3T3-E1 cells were observed for their adhesion and spreading morphologies. This assay was performed in triplicate.

### Cell proliferation assay

A total of 192 dried sterile SLA-Ti disks, each with a diameter of 5 mm, were divided evenly into six groups and placed on 96-well plates. Each group of titanium disks was soaked with SS, HP, CHX, RES, and NAR (100 µL/well) for 1 min, and the untreated SLA-Ti disks were used as the control. The disks were then rinsed twice with PBS. MC3T3-E1 (5 × 10^3^ cells per well) and L-929 cells (5 × 10^3^ cells per well) were separately seeded on the surface of rinsed titanium disks. After 1, 3, 5, and 7 days of culture, cell density on the surface of the titanium disks was quantified using the CCK-8 kit. After replacing the medium in each well, 10 µL of CCK-8 reagent was added and then incubated in the dark at 37°C for 2 hours. A microplate reader was used to read the absorbance of media at 450 nm. This assay was conducted with four wells per group in triplicate.

### Real-time quantitative PCR analysis

A total of 12 aseptic SLA-Ti disks measuring 30 mm in diameter were allocated into four groups and positioned on six-well plates. Each group was subjected to a 1 minute immersion in either SS, RES, or NAR or served as the control group with untreated SLA-Ti disks. The disks were then washed twice with PBS. MC3T3-E1 cells (5 × 10^4^ cells per well) were inoculated on the sample and then cultured for 7 days. RT-qPCR was used to assess the expression levels of osteogenic-related genes, like Runt-related transcription factors 2 (Runx2), Osterix (OSX), and Osteocalcin (OCN). The total RNA was isolated and purified with RNA Extraction Kit (Beyotime, China) and then reverse-transcribed into cDNA using Hiscript III Reverse Transcriptase (Vazyme, China). The RT-qPCR analysis was performed using ChamQ Universal SYBR qPCR Master Mix (Vazyme, China) together with cDNA. Three repetitions of the analysis were conducted, and GAPDH was used to normalize the data. The sequences of the primers are presented in Table 1.

**TABLE 1:** Primer sequences of target genes for RT-qPCR

Gene		Primer sequence (5’−3’)
Runx2	F	CCGAAATGCCTCCGCTGTTATG
	R	TCTGTCTGTGCCTTCTTGGTTCC
OSX	F	AGTTCACCTGCCTGCTCTGTTC
	R	GCGGCTGATTGGCTTCTTCTTC
OCN	F	CAAGCAGGAGGGCAATAAGGTAGTG
	R	CATACTGGTCTGATAGCTCGTCACAAG
GAPDH	F	GCTCTCCAGAACATCATCC
	R	TGCTTCACCACCTTCTTG

### Western blotting

The samples were processed in the same manner as described above. The expression levels of cellular proteins after different chemical decontamination were determined by western blotting. After being washed with PBS, the cells were lysed in radioimmunoprecipitation assay buffer containing phenylmethylsulfonyl fluoride to inhibit the protease enzyme. A polyvinylidene fluoride membrane (PVDF; Millipore, MA, United States) was employed to transfer the proteins obtained following SDS-PAGE separation. The membrane was then submerged in NcmBlot Blocking Buffer (New Cell & Molecular Biotech) for 20 min. The primary antibodies including Runx2 (12556, CST, USA), OSX (ab209484, Abcam, USA), OCN (ab93876, Abcam, USA), and GAPDH (60004, Proteintech, USA) were added to PVDF membrane for 12 h at 4°C. Following incubation, the membranes were washed with tris-buffered saline with 0.1% Tween 20 Detergent thrice and immersed in the secondary antibody (ZB-2301, ZSGB-BIO, China; AP124P, Millipore, United States) for 1 h at room temperature. An ECL Western Blotting Kit was used to generate protein signals, which were then imaged using the VILBER Multifunctional Imaging System (VILBER, Fusion FX, France). Internal control was provided by GAPDH.

### Statistical analysis

Statistical analyses were performed by Statistical Package for the Social Sciences (SPSS Inc., USA; version 22.0) and GraphPad Prism (GraphPad Software, USA; version 9.0). The comparisons were conducted using ordinary one-way analysis of variance (ANOVA) with Tukey’s multiple comparisons test. The examination of fluorescence images of live/dead bacteria, as well as the assessment of cell viability, was compared using a two-way ANOVA with a multiple comparisons test. A *P*-value of less than 0.05 indicated a statistically significant difference.

## RESULTS

### Surface characteristics changes

**Fig 1:**
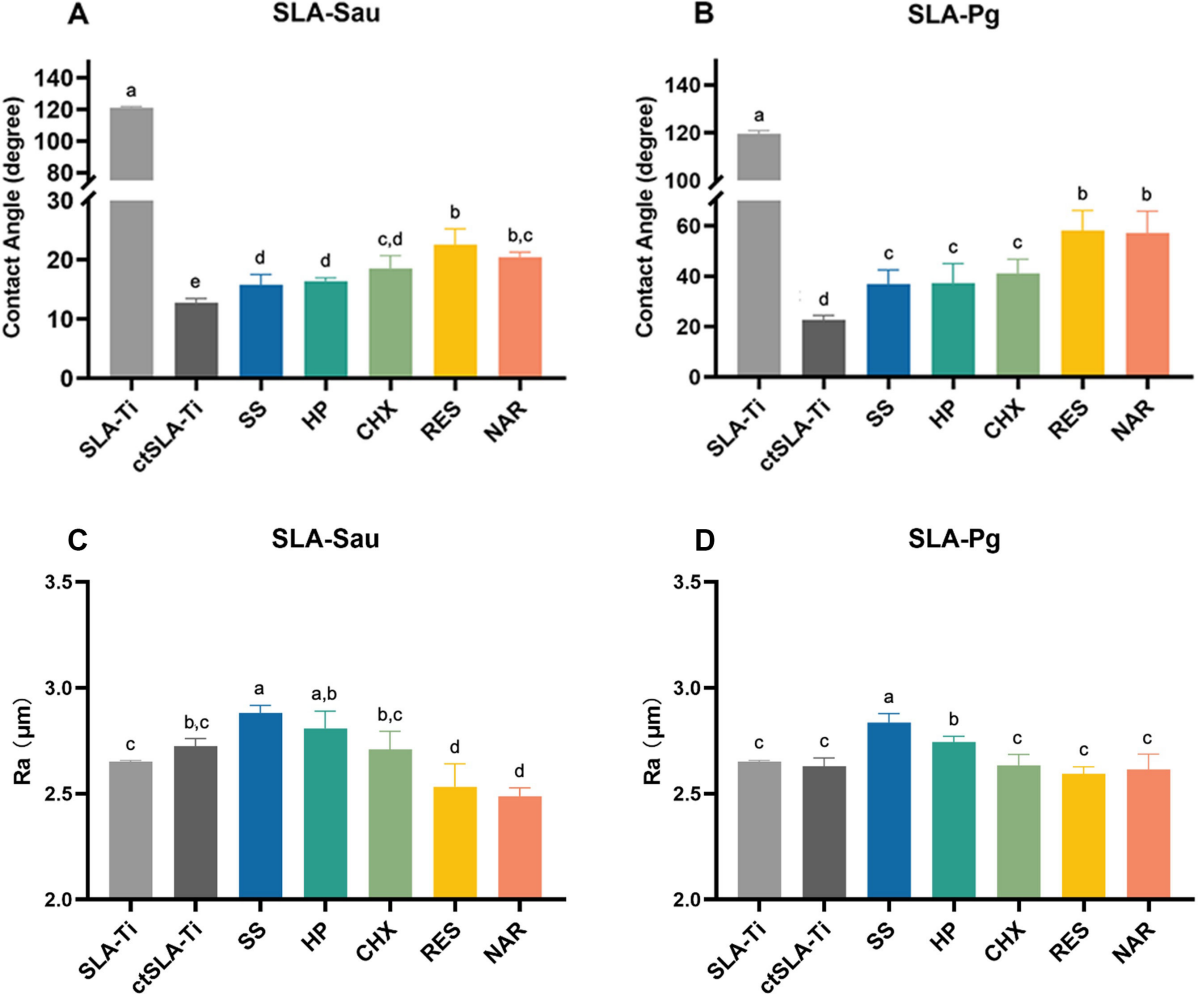
Wettability and roughness of titanium surfaces after chemical decontamination. (**A**) and (**C**) SLA-Sau; (**B**) and (**D**) SLA-Pg. SLA-Ti represents sandblasted and acid-etched titanium disks; ctSLA-Ti represents bacteria-contaminated SLA-Ti disks, including SLA-Sau or SLA-Pg. Results are presented as mean ± SD. Same lowercase letters indicate no significant difference in data between groups (*P* > 0.05), and different lowercase letters indicate significant differences in data between groups (*P* < 0.05).

The contact angle of titanium surfaces that were colonized by two distinct bacterial biofilms was determined after being rinsed with the different test chemical agents (Fig. 1A and B). SLA-Ti showed super-hydrophobicity than all the contaminated titanium disks. After contamination, titanium surfaces treated with RES and NAR showed greater contact angles compared to untreated surfaces and those rinsed with SS and HP. There was no statistically significant difference in the contact angles of CHX and NAR on SLA-Sau.

In addition, the roughness of SLA-Sau disks rinsed with SS and HP was significantly higher than uncontaminated titanium disks, while specimens rinsed with RES and NAR were significantly lower (Fig. 1C). Similar results can be observed on the surface of the SLA-Pg disks which RES and NAR exhibited reduced roughness compared to specimens rinsed with SS and HP, but no significant difference was observed with uncontaminated titanium disks (Fig. 1D). Among all the chemical agents, SS decontaminated samples exhibited the greatest surface roughness. It demonstrated that RES and NAR solutions effectively restored the wettability and may affect the roughness of titanium surfaces after decontamination.

### Micromorphological observation

**Fig 2:**
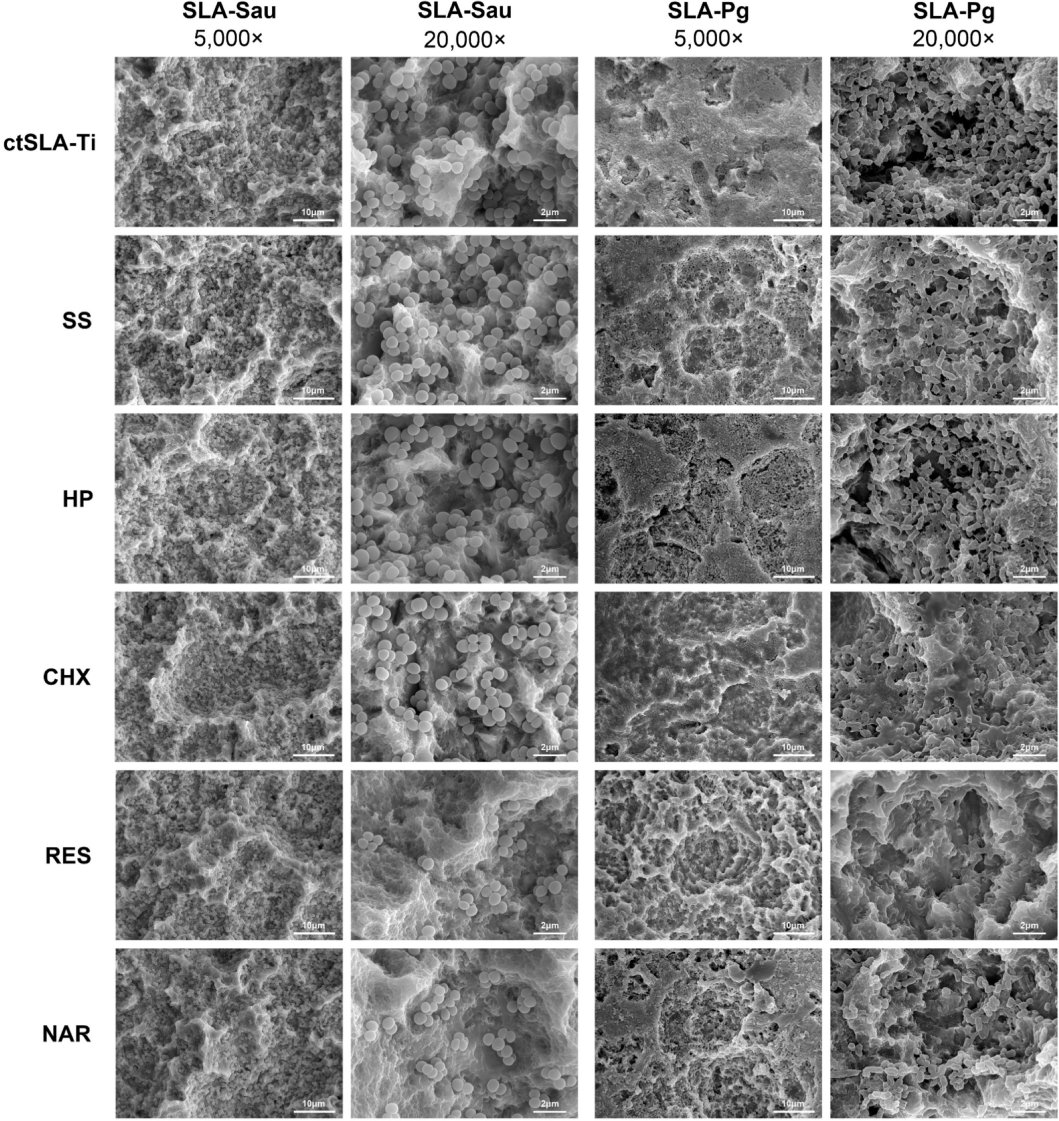
SEM images of biofilm destruction and reduction following various chemical decontaminations (magnification ×5,000 and ×20,000). ctSLA-Ti represents bacteria-contaminated SLA-Ti disks, including SLA-Sau or SLA-Pg.

SEM was used to observe surface morphology as a result of chemical decontamination (Fig. 2). Dense bacterial biofilms were visible on the surface of SLA-Sau and SLA-Pg. At low magnification, several types of disruptions, separations, and reductions of the surface biofilms may be seen when comparing samples decontaminated via various methods with bacteria-contaminated SLA-Ti disks (ctSLA-Ti). However, when examined at a higher magnification, no significant variations were observed in samples that underwent chemical decontamination using SS and HP. The bacterial biofilms on titanium disks rinsed with CHX, RES, and NAR were considerably reduced, which exposed a rough, cratered titanium substrate. Moreover, RES and NAR treatments were relatively more effective for SLA-Sau in comparison to CHX treatment. Similarly, both RES and NAR disrupted the biofilm integrity of SLA-Pg, whereas CHX decontamination resulted in a gelatinous appearance of the bacterial biofilm.

### Biofilm biomass assay

**Fig 3:**
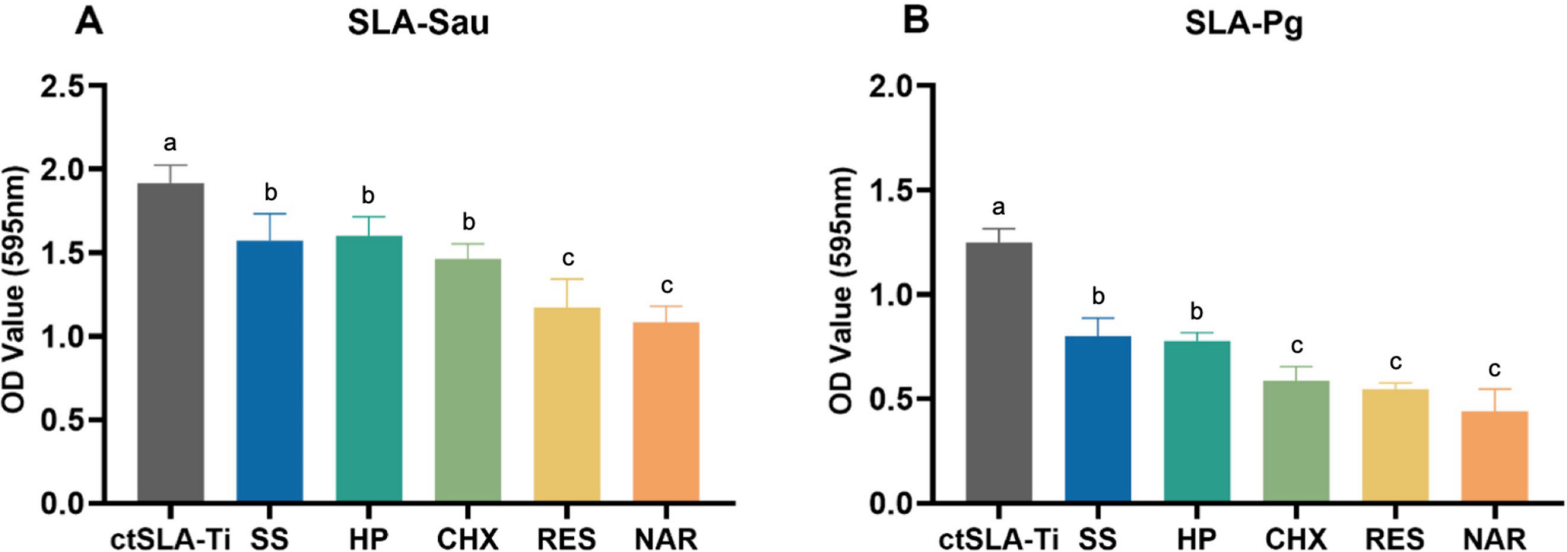
Quantitative biofilm biomass analysis using crystal violet staining after chemical decontamination. (**A**) SLA-Sau; (**B**) SLA-Pg. ctSLA-Ti represents bacteria-contaminated SLA-Ti disks, including SLA-Sau or SLA-Pg. Results are presented as mean ± SD. Same lowercase letters indicate no significant difference in data between groups (*P* > 0.05), and different lowercase letters indicate significant differences in data between groups (*P* < 0.05).

The remaining bacterial biofilms on titanium disks after chemotherapeutic cleaning were quantified via crystal violet staining procedure. All the treatments decreased the biofilm of both bacterial species. The reduction is more drastic after RES and NAR treatment for SLA-Sau and after RES, NAR, and CHX treatment for SLA-Pg. On the SLA-Sau surface (Fig. 3A), there was no significant difference in biofilm reduction between rinsing with SS, HP, and CHX, while both RES and NAR showed higher efficiency. For the SLA-Pg surface (Fig. 3B), RES and NAR also exhibited higher removal effects than SS or HP but were not significantly different from CHX.

### Live/dead bacteria staining and quantitative analysis

**Fig 4:**
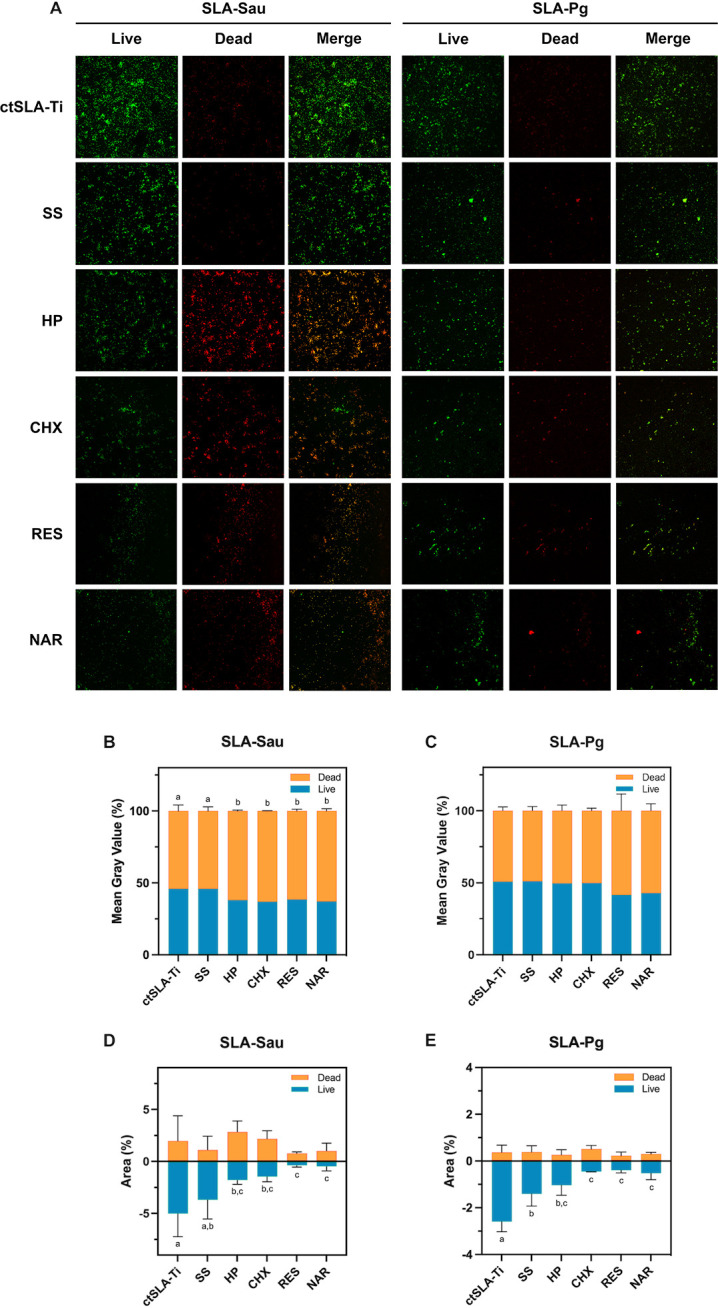
Quantitative composition of residual biofilms analysis using live/dead bacteria staining. (**A**) Fluorescence images of live/dead bacteria on the surface of different SLA-Ti disks after chemical decontamination (magnification ×200). (**B** and **D**) Mean fluorescence intensity and fluorescence area of live/dead bacteria in residual *S. aureus* biofilm. (**C** and **E**) Mean fluorescence intensity and fluorescence area of live/dead bacteria in residual *P. gingivalis* biofilm. ctSLA-Ti represents bacteria-contaminated SLA-Ti disks, including SLA-Sau or SLA-Pg. Data are presented as mean ± SD. Same lowercase letters indicate no significant difference in data between groups (*P* > 0.05), and different lowercase letters indicate significant differences in data between groups (*P* < 0.05).

Live/dead bacteria were visualized using fluorescence microscopy after distinct treatments on titanium disks (Fig. 4A). SS-rinsed samples presented a higher overall bacterial load containing a higher proportion of live bacteria. Conversely, the other four groups of samples treated with different test chemical rinses showed a significant reduction in the live bacterial proportions. CHX-, RES-, and NAR-treated groups significantly reduced live and dead bacteria.

The relative quantities of live and dead bacteria in each sample were assessed by quantitative image analysis, based on mean gray value and area measurements (Fig. 4B through E). The average fluorescence intensity of the remaining bacteria on the contaminated and SS-rinsed titanium disks was significantly greater for SLA-Sau and was dominated by live bacteria. HP did not reduce the total count of bacteria but relatively reduced the proportion of live bacteria, although no statistical difference was noted. In contrast, CHX, RES, and NAR removed more adherent bacteria from the sample surface and left fewer live bacteria. Similarly, CHX, RES, and NAR were found to be better decontaminators of SLA-Pg, although the fluorescence intensity did not show statistical significance.

### pH measurement and cytotoxicity

**Fig 5:**
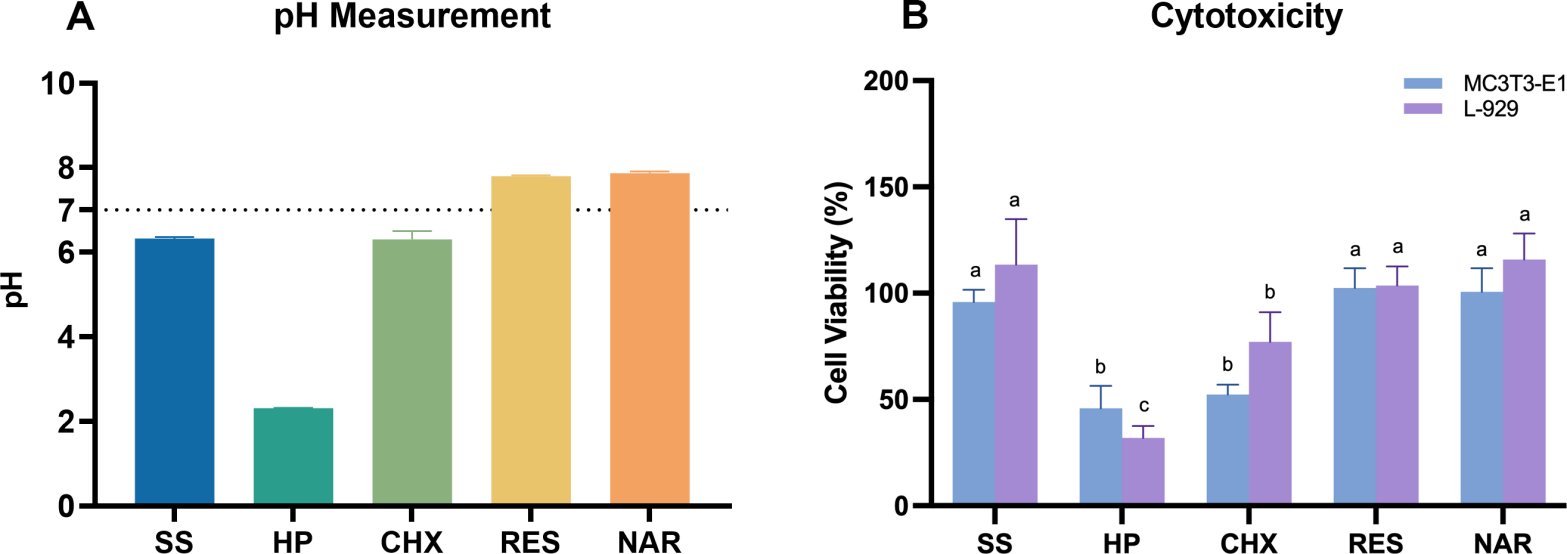
(**A**) pH value of various chemical decontaminations. (**B**) Cytotoxicity of different chemical decontaminations for MC3T3-E1 and L-929 cells. Data are presented as mean ± SD. Same lowercase letters indicate no significant difference in data between groups (*P* > 0.05), and different lowercase letters indicate significant differences in data between groups (*P* < 0.05).

The pH value of each solution varied according to its characteristics. Among them, HP was the most acidic, while RES and NAR were weakly alkaline (Fig. 5A). MC3T3-E1 and L-929 cells showed variability in cellular viability post-exposure to chemical agents (Fig. 5B). HP and CHX showed strong cytotoxicity to both types of cells. The viability of cells after RES or NAR treatment was similar to that of SS, that is, it showed no significant difference. In a word, for MC3T3-E1 cells, the order of cytotoxicity was HP = CHX < RES = NAR = SS, while it was HP < CHX < RES = NAR = SS for L-929 cells.

### Cell adhesion and spreading

**Fig 6:**
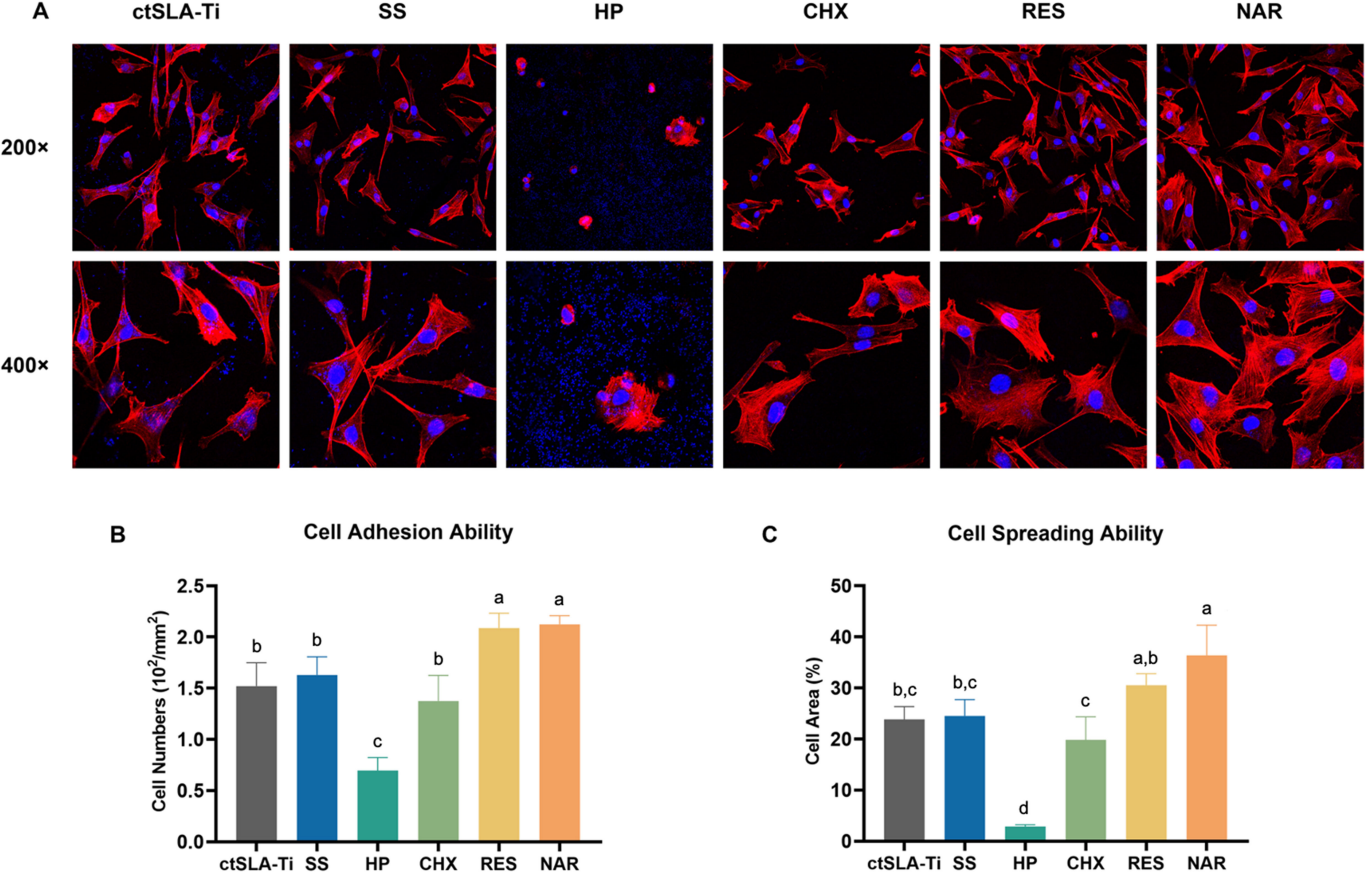
Adhesion and spreading of MC3T3-E1 cells on *S. aureus*-covered SLA titanium surfaces after chemical decontamination. (**A**) Confocal laser scanning microscopy images of MC3T3-E1 cells adhesion and spreading (magnification ×200 and ×400). (**B**) Statistical analyses of adhesive cell numbers and spreading area. ctSLA-Ti represents bacteria-contaminated SLA-Ti disks, including SLA-Sau or SLA-Pg. Data are presented as mean ± SD. Same lowercase letters indicate no significant difference in data between groups (*P* > 0.05), and different lowercase letters indicate significant differences in data between groups (*P* < 0.05).

The osteoblasts adhesion and spreading were observed by LSCM after chemical decontamination and incubation with *S. aureus* for 4 hours (Fig. 6). After soaking the titanium disks in RES and NAR, an improved cell morphology was detected on the surface, in comparison to untreated disks or those soaked in SS, HP, and CHX. Increased cell adhesion was observed, characterized by a more distinct cytoskeleton and higher levels of extended pseudopodia. Furthermore, the CHX, RES, and NAR groups revealed a reduced presence of bacterial nuclei in the background. It should also be noted that the HP treatment attenuated cell adhesion in MC3T3-E1 cells which contracted in clumps on the titanium surface compared to other groups. Based on the nuclei quantification, more osteoblasts adhered to the surface after RES and NAR treatment, whereas HP treatment significantly reduced the cell count. Consequently, the samples’ surfaces treated with HP and CHX were not conducive to osteoblast adhesion.

### Cell proliferation

**Fig 7:**
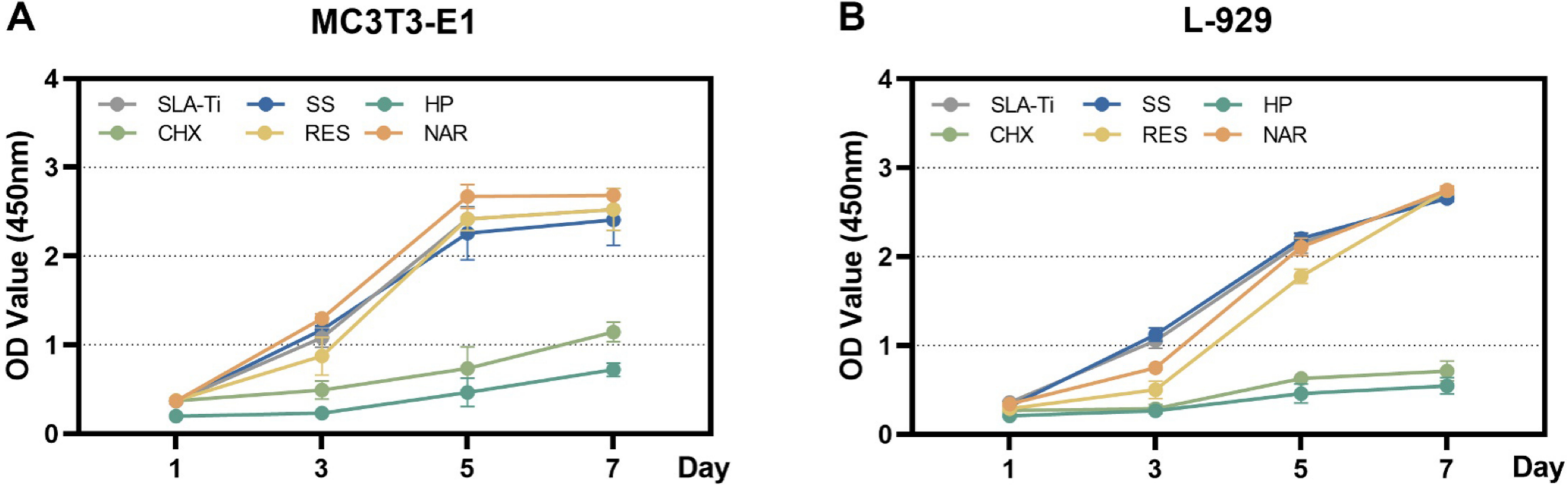
Cell proliferation of MC3T3-E1 cells and L-929 cells on SLA titanium disks exposed with chemical decontaminants after 1, 3, 5, and 7 days in culture. (**A**) MC3T3-E1 cells. (**B**) L-929 cells. Data are presented as means ± SD.

A CCK8 assay was used to determine whether osteoblasts and fibroblast proliferation were affected by chemical decontaminants on SLA-Ti disks (Fig. 7). After culturing for 1 day, when the titanium surface was rinsed with various solutions, only the treatment with HP significantly suppressed the proliferation of osteoblasts. The other groups showed similar cell densities. However, the osteoblasts and fibroblasts in the CHX group also demonstrated decreased levels of proliferation after 3 days of culture, similar to the HP group. Therefore, while the number of cells on all titanium surfaces steadily grew, the CHX and HP groups exhibited reduced rates of growth for both types of cells. Cell proliferation in the SS group was similar to that of the control group. Cells in the RES and NAR groups revealed accelerated proliferation rates starting from day 3 and ultimately achieved comparable levels of proliferation by days 5 and 7 of the culture period. As a result, the growth of MC3T3-E1 and L-929 cells was hindered by the chemical decontaminants HP and CHX. On the other hand, treatments with RES and NAR stimulated a rise in cell density.

### Osteoblast differentiation

**Fig 8:**
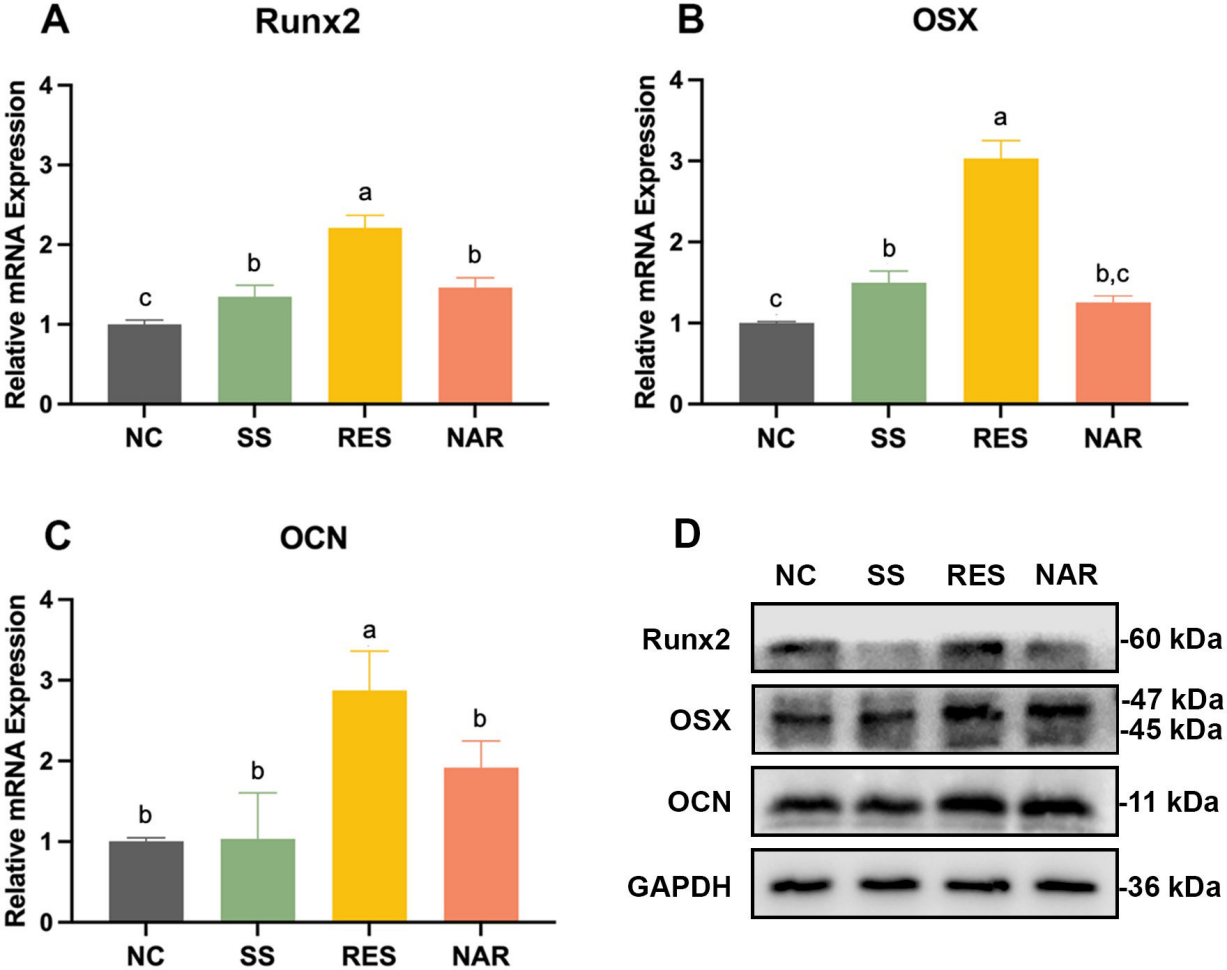
Osteogenic differentiation of MC3T3-E1 cells with SS, RES, and NAR rinsing after 7 days in culture. (**A**) mRNA expression of Runx2. (**B**) mRNA expression of OSX. (**C**) mRNA expression of OCN. (**D**) Protein expressions of Runx2, OSX, and OCN. Data are presented as means ± SD. Same lowercase letters indicate no significant difference in data between groups (*P* > 0.05), and different lowercase letters indicate significant differences in data between groups (*P* < 0.05).

RT-qPCR was used to measure osteoblast-specific mRNA marker expression following SS, RES, and NAR treatment. Osteogenic differentiation and regeneration are regulated by key markers, like Runx2, OSX, and OCN, which are involved in mineralization (22). RES-treated groups showed greater mRNA expression on the titanium surface compared to untreated groups (Fig. 8A through C). Western blot analysis was used to determine the expression of osteoblast marker proteins after treatments with SS, RES, and NAR. The RES treatment group showed the highest protein expression markers on the titanium surface (Fig. 8D). Through western blot analysis, it was further validated that the RT-qPCR results emphasized the potential role of RES in promoting osteoblast differentiation and bone integration.

## DISCUSSION

Removing plaque with chemical decontamination is an important method for treating peri-implant disease. However, plant extracts have rarely been studied for their ability to remove mature plaque from rough titanium surfaces. In the current research, the removal efficiency of mature bacterial biofilms and the cytocompatibility of RES and NAR solutions on SLA-Ti surfaces were analyzed, in comparison to the commonly used chemical decontaminants. The findings obtained showed that solutions of RES and NAR efficiently decreased the biofilm biomass on rough titanium disks, restored the surface characteristics of the implants, and neutralized the acidic microenvironment. In addition, RES and NAR solutions display lower cytotoxicity and promote the restoration of biocompatibility on the titanium surface, as compared to HP and CHX.

Evidence has suggested that bacteria are capable of inducing electrochemical corrosion on metal surfaces and causing damage to the oxide layer, resulting in alterations to surface properties (23). Therefore, recovering the surface properties after the removal of the bacterial plaque is essential for implant osseointegration (24). The SEM scans revealed that bacteria adhere to the titanium surface and cluster together in various locations, eventually developing into a unified microbial film. After chemical debridement, the integrity and continuity of the bacterial plaques were disrupted. This resulted in CHX, RES, and NAR solutions being more effective in disrupting and reducing bacterial plaque adhesion, thereby restoring a larger surface area to SLA-Ti. The eradication of bacteria took place not only on the exterior layer but also proved to be as efficient in eliminating bacteria that were attached to the inner crevices.

Furthermore, the presence of bacterial biofilm greatly enhances the wettability of the SLA-Ti as a result of the microstructure of the substrate being occupied by bacteria (25, 26). After treatment with RES and NAR solutions, the contact angle of the titanium surface can be significantly restored, which more closely resembles the surface properties of clean SLA-Ti disks. There is a possibility that a fully developed layer of plaque was removed from the surface, revealing the underlying hydrophobic titanium substrate. In addition, the elimination of plaque reinstates the rough texture of the titanium surface (27). Surface roughness was positively correlated with bacterial adhesions (28, 29). RES and NAR solutions decreased the roughness of the *S. aureus*-covered titanium surface, while it was not significant for *P. gingivalis*-covered titanium surface. Such difference may be due to the variation in cell wall peptidoglycan content between Gram-positive and Gram-negative bacteria, and the morphological differences resulting in different adhesions (11, 30, 31).

As a further step, the remaining biofilm and the distribution of live or dead bacteria on the titanium surface were quantified following chemical decontamination (Fig. 4). In the case of *S. aureus*, it was observed that treatment with SS had no impact on the quantity and nature of the plaques. However, treating with HP resulted in a higher percentage of dead bacterial cells within the residual plaque without affecting the overall amount of attached plaque. These remaining live bacteria can be detrimental to the peri-implant microenvironment. Nevertheless, both SS and HP were able to remove part of the *P. gingivalis* biofilm, and the cleaning effect did not differ significantly between them. Consistent with the previous studies, saline did not exhibit much antimicrobial activity, likely due to the physical effect of polishing on biofilms (32). HP demonstrated the capacity to eradicate bacterial biofilm by the oxidation of certain cellular constituents and the generation of gas, hence offering distinct benefits in combating biofilm formed by anaerobic bacteria such as *P. gingivalis* (33). In addition, CHX, a broad-spectrum antibacterial agent, is one of the most commonly used sterilizers and is recognized as the gold standard among chemical decontaminants in clinical practice. It showed moderate efficacy in eliminating biofilms of both bacteria (34).

For mature bacterial biofilms on SLA titanium disks, both RES and NAR solutions showed better removal than SS and HP, showing less plaque residue and live bacterial ratios. Furthermore, the NAR solution has shown superior efficacy in eliminating *S. aureus* compared to CHX. RES and NAR have been found to interfere with quorum sensing (QS) in bacteria, which are a gene regulatory mechanism that influences the formation of bacterial aggregation and the development of biofilms (35–37). Secreted peptides acted as autoinducers during the QS of *S. aureus* (35). Disruption of the QS system can be caused by RES interfering with the production of surface proteins and capsular polysaccharides (38), whereas NAR can directly decrease the amounts of cell-bound peptides and polysaccharides (37). Moreover, NAR has the potential to disrupt the gene expression of phenol-soluble modulins, which are directly controlled by accessory gene regulators. This can result in changes to the surface activity of *S. aureus* (39–41). In contrast, acyl homoserine lactones (AHLs) are signals involved in the process of QS for *P. gingivalis* (42, 43). These AHLs regulate the production of rhamnolipid, a kind of surfactant molecule. This leads to the generation of channels in mature biofilms and an increase in the shedding of bacteria (44, 45). Specifically, RES can interfere with the synthesis of rhamnolipid and alter the surface tension of bacteria. NAR can antagonize *LasR* and *RhlR* receptors and block the signaling of AHLs, leading to the isolation of biofilms (46).

Second, extracellular polymeric substances (EPSs) constitute the major component of bacterial biofilm, which consists of polysaccharides, proteins, and extracellular DNA (eDNA) (47, 48). The disruption of EPS facilitates the penetration of antibacterial drugs into plaques and aids in the detachment of biofilms. A research study has demonstrated that polyphenolic chemicals can modify the properties of bacterial cell membranes, including their electrical charge and cellular osmotic pressure. These modifications can lead to the formation of localized ruptures or holes in the membrane, causing the leakage of cellular contents (49). RES may also contribute to bacterial cell membrane rupture through site-specific oxidative damage (50). Likewise, flavonoids damage bacterial cell membranes, reduce eDNA levels, and disrupt electrostatic interactions and polysaccharide adhesion between bacteria (48, 51, 52). Furthermore, RES and NAR may decrease bacterial swarming motility and swimming motility to hinder bacterial attachment, which is crucial for preventing bacterial biofilm re-formation and the preferred colonization of decontaminated titanium surfaces by host cells (38, 53).

The average pH value within a healthy human oral cavity is 7.27, which represents a relatively neutral environment conducive to oral health (54). The proliferation of microorganisms results in the generation of acids, which cause a significant reduction in the pH levels surrounding titanium implants (12, 55), and acidic environments can corrode and destroy metal oxide layers (56). Besides the impact of bacteria, the chemical decontaminants themselves can modify the surface qualities of the material (32). The destruction of the titanium surface oxide layer is directly related to the acidity of the decontaminant used (11). The pH values of different chemotherapeutic agents were measured (Fig. 5A). Both RES and NAR solutions exhibited weak alkalinity, which can counteract the acidic byproducts generated by bacteria during their metabolic processes. Consequently, this diminishes the pace at which bacteria may cause corrosion to metals.

Biocompatibility is a crucial factor influencing the re-osseointegration on the titanium surface after decontamination. Cell adhesion is a crucial factor in cell growth and differentiation, serving as the initial interaction between host cells and biomaterials. Bacteria engage in “surface competition” with host cells on contaminated implants, therefore impacting the adherence of the cells (57). The dominant factor in the competition determines the re-osteointegration of the implant or secondary infectious dislodgement. In this study, MC3T3-E1 cells were inoculated on the surface of decontaminated titanium disks and co-cultured with *S. aureus* to observe the early adhesion of the cells. The cells on the surfaces of the samples treated with peroxide formed clusters and displayed the most unfavorable adhesion condition. An investigation has proven that HP induces an elevation in the levels of intracellular reactive oxygen species (ROS) in MC3T3-E1 cells, resulting in oxidative damage (58). In addition, CHX reduces the number of osteoblast adhesions, which is consistent with previous study (32). CHX suppresses the production of collagen in osteoblasts, and this suppression of cells may have a broad impact due to its strong cationic nature (59)

RES and NAR, however, enhanced osteoblast adhesion to *S. aureus*-contaminated titanium surfaces. As an important determinant, the virulence factor of bacteria plays a key role. RES has been found to reduce the activity of hemolysin, a key virulence component of *S. aureus* that disrupts the phospholipid bilayer structures of host cell membranes. This reduction is achieved by suppressing the expression of the *saeRS* gene, as well as inhibiting the activity of lipase, another virulence factor that damages host cell membranes (60–62). By reducing the production of extracellular virulence enzymes, NAR can inhibit bacterial virulence factors (37). Moreover, these two decontaminants also show similar effects in reducing the virulence of *P. gingivalis*. RES can attenuate bacterial virulence by inhibiting the gene expression of bacterial hairs and gingival proteases (63). *P. gingivalis* releases proteases that facilitate the breakdown of collagen in gingival epithelial cells, whereas RES may impede the degradation of extracellular matrix proteins (64). Likewise, rinsing with flavonoids can destroy bacterial flagella and bacterial hair structures (51). Moreover, RES and NAR may decrease bacterial clustering and adhesion, thereby reducing infection (64).

Chemical agents have an impact on both bacterial biofilms and peri-implant tissues, as well as healthy oral mucosa, during the process of decontamination. Hence, the cytotoxicity of the studied chemical decontaminants on cells resembling osteoblasts and fibroblasts was also assessed (Fig. 5B). Both HP and CHX exhibited potent cytotoxicity, with approximately half of the healthy cells dying after 1 min of exposure to the solution. In contrast, neither RES nor NAR solutions showed cytotoxicity to osteoblasts and fibroblasts compared to SS. Moreover, the solute may adhere to the titanium dioxide surface either by electrostatic adsorption or non-covalent bonding in the washing process and may not be fully eliminated even with thorough rinsing. CHX displays quick adsorption onto the titanium surface, achieving equilibrium within 60 seconds and thereafter undergoes slow dissociation. Rinsing alone is insufficient to remove the adsorbed CHX (65). Furthermore, to evaluate the impact of residual decontaminants on cell proliferation, osteoblastic and fibroblastic cells were further introduced onto titanium disks that had been immersed in various solutions. As shown in previous studies, HP and CHX treatment inhibited cell growth, which was associated with their pro-oxidability and cytotoxicity (32, 58). HP decreases cell survival by inducing oxidative stress, resulting in DNA damage and membrane rupture (58). Conversely, CHX elevates intracellular levels of ROS, leading to the depletion of glutathione and worsening oxidative damage to cells (66).

Similar to the ability of cells to adhere, both RES and NAR enhanced cell proliferation and encouraged the differentiation of osteoblasts, consistent with prior findings (67). RES stimulated the proliferation and differentiation of osteoblasts, which had a role in the regeneration of bone tissue in peri-implant diseases (68). It has been shown that NAR also increases the expression of osteoblast protein markers, including osteoprotegerin, osteopontin, bone morphogenetic protein, and OCN. This leads to an enhancement in osteoblastic activity and bone mineral density (69–71). Moreover, both RES and NAR significantly increased the fibroblast proliferation. It has been found that RES stimulates the production of collagen III and improves the proliferation and migration of human gingival fibroblasts (72). Oxidized NAR exhibited good cytocompatibility and could promote the secretion of growth factors by fibroblasts (73).

The limitations of the current study are similar to previous studies. The application of the single debridement method was not able to altogether remove the biofilm and obtain the desired treatment results (10). While *S. aureus* and *P. gingivalis* can be found on the surfaces of infected implants, it is important to note that exposing a single bacteria does not accurately replicate the actual pathogenic environment. While bacterial adhesion to titanium surfaces has been a valuable model for *in vitro* investigations, it fails to effectively simulate the intricate and fluctuating conditions in the mouth cavity, as well as the impact of external mechanical forces. Moreover, the operational mechanism of these two chemical decontaminants necessitates further empirical investigation. We plan to continue our exploration in this direction such as actively test the application potential of the cocktail combining both RES and NAR.

### Conclusions

This study first investigated the efficacy and biocompatibility of RES and NAR solutions as chemical decontaminants in the debridement of mature bacterial biofilms from SLA-Ti surfaces. These two agents could restore titanium surface properties, remove bacterial biofilm, and have better biocompatibility. Although complete removal of formed biofilms cannot be achieved, their clinical application as chemotherapeutic agents intended to prevent and treat implant-associated infections still holds a wide range of promise.
